# Land Degradation Changes the Role of Above- and Belowground Competition in Regulating Plant Biomass Allocation in an Alpine Meadow

**DOI:** 10.3389/fpls.2022.822594

**Published:** 2022-02-03

**Authors:** Yong Zhang, Qiuzhu Zheng, Xiaoxia Gao, Yandan Ma, Kemin Liang, Haitao Yue, Xiaoxia Huang, Kaiting Wu, Xiaorong Wang

**Affiliations:** ^1^Yunnan Key Laboratory of Plateau Wetland Conservation, Restoration and Ecological Services, College of Wetlands, Southwest Forestry University, Kunming, China; ^2^National Plateau Wetlands Research Center, Southwest Forestry University, Kunming, China; ^3^State Key Laboratory of Vegetation and Environmental Change, Institute of Botany, Chinese Academy of Sciences, Beijing, China; ^4^School of Earth Sciences, Yunnan University, Kunming, China

**Keywords:** land degradation, aboveground traits, root competition, R/S ratio, alpine meadow, eastern Qinghai-Tibetan Plateau

## Abstract

The allocation pattern of plant biomass presents the strategy of the plant community to adopt environmental changes, while the driver of biomass allocation is still unclear in degraded alpine grassland ecosystems. To explore the issue, this study investigated the shoot-to-root (R/S) ratio, plant aboveground traits, and root competition of three functional groups (i.e., grasses, sedges, and forbs) at three degradation levels (i.e., no obvious degradation, ND; moderate degradation, MD; and severe degradation, SD) in an alpine meadow in the eastern Qinghai-Tibetan Plateau. The relationships among plant aboveground traits, root competition, and R/S ratio were tested using the structural equation model (SEM). The results showed that the shoot and root biomass tended to decrease, but the R/S ratio of the plant community did not change along the degradation gradient. Plant height, lateral spread, and leaf length of most plant functional groups reduced, while leaf width and leaf area of most plant functional groups did not change along the degradation gradients. The root competition ability (presented as the fraction of root biomass in total biomass) of sedges in MD was the lowest, while that of grasses was the highest. The effects of aboveground competition on the R/S ratio were non-linear because of the different roles of plant height, lateral spread, and leaf area in regulating the R/S ratio along the degradation gradient. In contrast, the effects of belowground competition on the R/S ratio were linear because belowground competition promoted the R/S ratio, and the strength of this effect reduced along the degradation gradient. These results indicate that plant competition might be a critical factor to maintain the high R/S ratio in degraded alpine meadows.

## Introduction

According to the Food and Agriculture Organization of the United Nations, the grasslands and woodland ecosystems cover nearly 35% of the terrestrial area around the world (Dubois, 2011). The grassland ecosystem has a crucial role in global carbon exchange because of its high carbon storage (Scurlock and Hall, 2002). Especially, the biomass allocation of plants [i.e., the shoot-to-root (R/S) ratio] is an important factor for carbon modeling in terrestrial ecosystems (Poeplau, 2016). The R/S ratio of plant community in the grassland ecosystem is widely discussed (Donald, 1958; Wilson, 1988; Yang et al., 2018). Generally, the root biomass is higher than the shoot biomass in grassland ecosystems (Dai et al., 2020). The increase in the R/S ratio could promote the stability of the grassland ecosystem (Tilman et al., 2006) and indicate the response of the plant community to environmental stresses (Agathokleous et al., 2018). Usually, environmental stress (e.g., intense herbivory and drought) could lead to an increase in the R/S ratio (McNaughton et al., 1998; López–Mársico et al., 2015; Wilson et al., 2018; Alon et al., 2019; Hao and He, 2019). The availability of nutrients and water (Wang et al., 2018) or a restoration process (Gao et al., 2021), however, could reduce the R/S ratio of the plant community. Therefore, the increase in the R/S ratio might indicate that the habitat of the plant community gets worse. Habitat deterioration could change the species richness of the plant community. The change in species richness will inevitably shift the competition pattern among plant species (Herben et al., 2003).

The competition, which could be described as the disequilibrium of biomass among species, is a primary perspective to explain the construction of the plant community (Wilson, 1988; Tilman, 2020). Both the aboveground competition and belowground competition are always positively correlated but they play different roles in shaping the R/S ratio because the aboveground competition of plants focuses on the snatch of light resources, while the belowground competition of plants focuses on the uptake of nutrients and water (Donald, 1958). Obviously, larger shoot biomass will lead to a lower R/S ratio, and larger root biomass will lead to a higher R/S ratio (Pugnaire and Luque, 2001). But the effects of plant competition on plant biomass remain unclear. The controlled experiment suggested that the plant biomass is positively correlated (Bonser and Reader, 1995) or did not correlate (Schofield et al., 2019) with plant competition intensity. However, some field investigations suggested a negative correlation between them (Nötzold et al., 1997).

The Qinghai-Tibetan Plateau (QTP), which is also called the third pole of the world, is the largest alpine region around the world and is mostly occupied by alpine meadows (Dong et al., 2020). In recent decades, these alpine meadows are suffering serious degradation due to climate changes and inappropriate anthropic disturbance (Cao et al., 2019). Land degradation increased the R/S ratio of alpine meadows in the QTP due to a significant decrease in aboveground biomass and a weak change in belowground biomass (Zhang et al., 2019). Moreover, the negative effects of land degradation on plant species richness, plant composition, and plant competition have been widely studied in the QTP (Wang et al., 2014; Tang et al., 2015; Luo et al., 2020; Peng et al., 2020). However, it is still unclear whether the land degradation would change the role of above- and belowground competition in regulating the R/S ratio. To explore this issue, we conducted a field survey in an alpine meadow with different degradation gradients in the eastern QTP. We hypothesized the following: (1) The aboveground competition would reduce, while the belowground competition would promote the R/S ratio, and (2) the effects of above- and belowground competition on the R/S ratio would decrease along the degradation gradient.

## Materials and Methods

### Study Site

**FIGURE 1:**
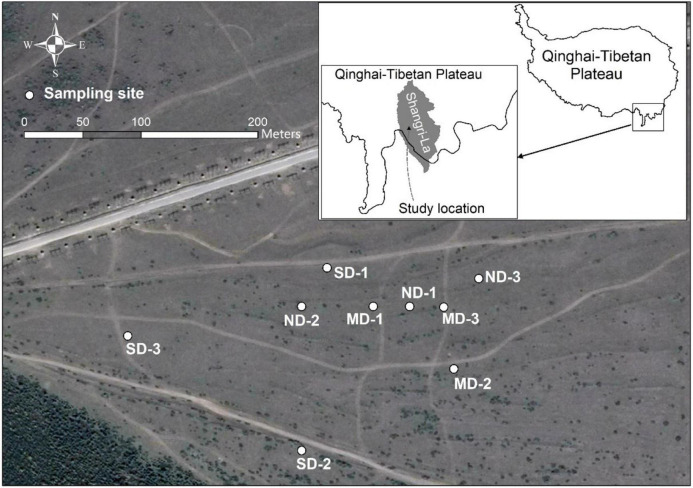
The location of the study area and the distribution of sampling sites.

This study was conducted on an alpine meadow in Shangri-La (27°47.992′ N, 99°39.094′ E, 3,200∼3,350 m a.s.l.), Yunnan Province, China (Figure 1). Shangri-La, located in the eastern QTP, distributes nearly 1,400 km^2^ alpine meadows. These meadows maintain the development of local communities by supporting tourism and livestock husbandry. The annual precipitation is 619.5 mm, and the annual temperature is 6.9°C (2,000∼2,016) in Shangri-La. Tourist trampling and significant warming trend have been leading to alpine meadow degrading in this region (Huang et al., 2015; Lehnert et al., 2016).

Three degradation levels (i.e., ND, MD, and SD) were identified according to the width of tourism trails and plant coverage, plant height, soil bulk density, and water content of surface soil aside from the road (10 cm aside the edge of the trial) (Table 1). The primary species of grasses were *Elymus burchan-buddae*, *Eragrostis nigra*, and *Poa annua*. The primary species of sedges were *Kobresia duthiei*, *Kobresia pygmaea*, and *Carex enervis*. For forbs, the primary species were *Saussurea romuleifolia*, *Stellera chamaejasme*, *Iris ruthenica var. nana*, and *Potentilla*. The grasses, sedges, and forbs varied among these three degradation levels (Supplementary Figure 1).

**TABLE 1:** The width of tourism trails (WTT), plant coverage (PC), plant height (PHt), soil bulk density (SBD), and water content of surface soil (WCSS) in no obvious degradation (ND), moderate degradation (MD), and severe degradation (SD) plots in this study (*n* = 27, α = 0.05).

Degradation level	WTT (m)	PC (%)	PHt (cm)	SBD (g/cm^3^)	WCSS (%)
ND	No trail	89.72 ± 0.68a	11.55 ± 0.53a	0.68 ± 0.04b	54.27 ± 3.09a
MD	1.8∼1.9	77.89 ± 1.33b	8.44 ± 0.27b	0.70 ± 0.05b	47.56 ± 3.16a
SD	2.5∼3	63.00 ± 2.06c	5.81 ± 0.25c	0.97 ± 0.05a	31.41 ± 1.84b

*Different lowercase letters mean significant differences among degradation gradients.*

### Field Sampling and Sample Handling

#### Collecting Data on Plant Aboveground Morphological Traits

Along the degradation gradients, 27 1 m × 1 m investigation quadrats (three degradation levels × three repetitions at each level × three parallels at each repetition) were conducted in July 2018. In each quadrat, the aboveground portion of the dominated plant individuals was collected at each sampling quadrat to measure the height, lateral spread, leaf length, leaf width, and leaf area of meadow plants. In total, 627 individuals (including 183 individuals of grasses, 147 individuals of sedges, and 297 individuals of forbs) were measured (Table 2). The plant height reflected the vertical competition potential of plants, while the lateral spread and leaf area reflected the horizontal competition potential of plants.

**TABLE 2:** The species and number of plant individuals that were collected in no obvious degradation (ND), moderate degradation (MD), and severe degradation (SD) plots.

	Grasses	Sedges	Forbs
ND	*Elymus burchan-buddae* (21) *Eragrostis nigra* (22)	*Kobresia duthiei* (22) *Kobresia pygmaea* (14)	*Saussurea romuleifolia* (22) *Stellera chamaejasme* (5) *Potentilla* spp. (21) *Iris ruthenica var. nana* (24)
MD	*E. burchan-buddae* (35) *E. nigra* (30) *Poa annua* (5)	*Carex enervis* (5) *K. duthiei* (34)	*Potentilla* spp. (45) *S. romuleifolia* (5) *Leontopodium franchetii* (25) *S. chamaejasme* (5) *Lomatogonium carinthiacum* (5) *I. ruthenica var. nana* (30)
SD	*E. burchan-buddae* (25) *E. nigra* (45)	*C. enervis* (18) *K. duthiei* (45) *K. pygmaea* (9)	*Plantago major* (5) *Potentilla* spp. (53) *S. romuleifolia* (2) *L. franchetii* (15) *I. ruthenica var. nana* (35)
	183	147	297

#### Collecting Data on the Shoot and Root Biomass

**FIGURE 2:**
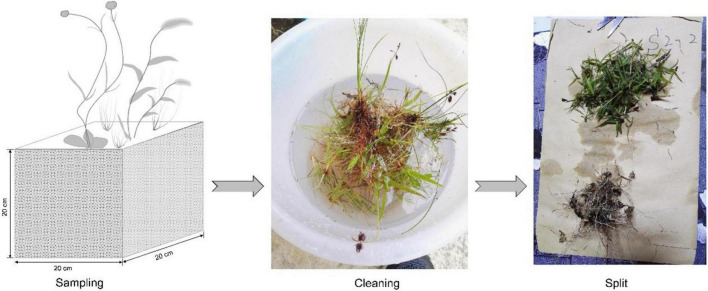
Collection of root and shoot biomass.

According to a pre-survey in this study area, we found that the distribution depth of most plant roots is less than 20 cm in the soil. Alongside each 1 m × 1 m investigation quadrat, a soil cube with a size of 20 cm × 20 cm × 20 cm carrying aboveground biomass was collected to obtain shoot and root biomass. The collected soil cubes were washed four times to remove all soil and dead roots. The cleaned individuals were separated into different functional groups (i.e., grasses, sedges, and forbs). Each individual was split into aboveground and belowground sections (Figure 2). These aboveground and belowground materials were dried and weighted to obtain the shoot and root biomass, respectively. Then, the R/S ratio was calculated according to the root and shoot biomass at the functional group level. The root competition was defined as the proportion of root biomass among different functional groups, i.e., for a functional group, a higher proportion of root biomass means a stronger root competition potential.

### Statistical Analysis

One-way ANOVA was conducted to test the variance of R/S ratio, root biomass, and morphological traits (including height, lateral spread, leaf length, leaf width, and leaf area) among degradation levels. Tukey’s *post hoc* test was conducted at the level of α = 0.05. Linear regression was conducted to reveal the relationship between aboveground biomass, belowground biomass, and the R/S ratio. The relationship between height and lateral spread was tested using regression analysis. The one-way ANOVA and regression analysis were conducted in R3.5.1.

The structural equation model (SEM) was used to test the direct and indirect effects of plant aboveground morphological traits (i.e., plant height, lateral spread, and leaf area) on the R/S ratio. We hypothesized that the vertical advantages (i.e., the height) and horizontal advantages (i.e., the lateral spread and leaf area) could directly affect the R/S ratio; meanwhile, they could indirectly affect the R/S ratio by changing root competition. According to this hypothesis, we constructed an *a priori* model that contained all potential paths (Supplementary Figure 2). The exogenous variables were plant height, lateral spread, and leaf area. The endogenous variables were root competition and R/S ratio. The model was optimized by stepwise removal of non-significant paths in ND, MD, and SD (Supplementary Tables 1–3). The qualified model was symbolized by a non-significant chi-square test (*p* > 0.05), low root mean square error of approximation (RMSEA < 0.05), high comparative fit index (CFI > 0.95), high Tucker-Lewis index (TLI > 0.95), low Akaike information criterion (AIC) value, and high squared multiple correlations (SMC) of endogenous variables. The SEM was conducted in IBM SPSS AMOS 24.

## Results

### Effects of Land Degradation on Plant Biomass Allocation

**FIGURE 3:**
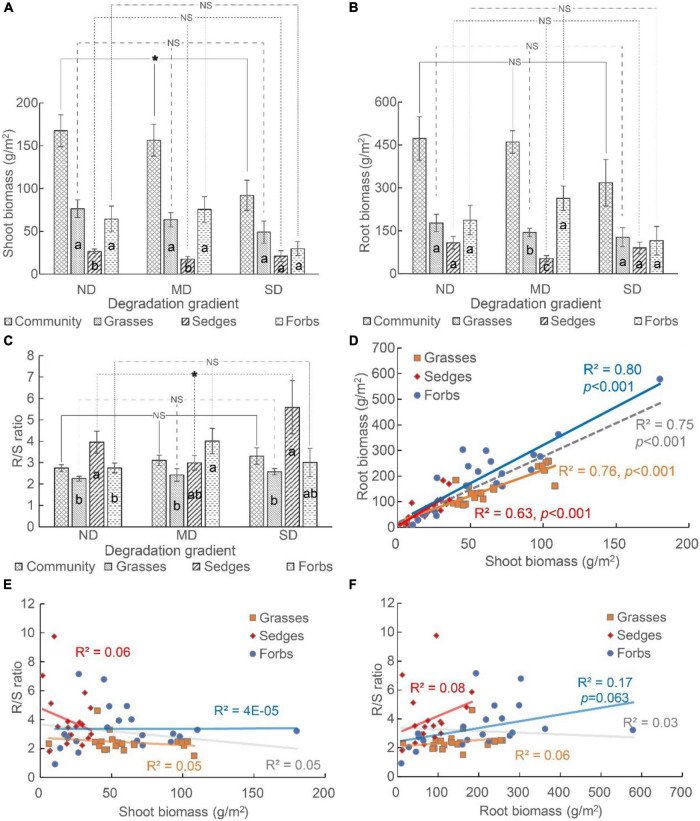
The **(A)** shoot biomass, **(B)** root biomass, and **(C)** root-to-shoot ratio (i.e., R/S ratio) of plant community and different functional groups (i.e., grasses, sedges, and forbs), and **(D–F)** the relationship among shoot biomass, root biomass, and R/S ratio. The “*” means the significant difference among functional groups along the degradation gradient. NS means no differences were detected. The different lowercase letters mean significant differences among functional groups in each degradation gradient.

Land degradation reduced the shoot biomass but did not affect the root biomass of the plant community. At the functional group level, land degradation did not change the shoot biomass and root biomass of grasses, sedges, and forbs (Figures 3A,B).

The biomass distribution among functional groups varied among different degradation levels. The shoot biomass of grasses and forbs was higher than that of sedges in ND and MD, while the shoot biomass did not vary among these three functional groups in SD (Figure 3A). The root biomass did not vary among different functional groups in ND and SD. The order of root biomass was forbs > grasses > sedges in MD (Figure 3B).

The R/S ratio was >2 in all degradation conditions (Figure 3C). The R/S ratio of the plant community, grasses, and forbs did not differ among ND, MD, and SD (Figure 3C). For sedges, the R/S ratio was the lowest at MD, and it was the highest at SD (Figure 3C). The R/S ratio of different functional groups varied at each degradation gradient. In ND and SD, the R/S ratio of sedges was the highest. In MD, the R/S ratio of forbs was the highest (Figure 3C).

The shoot biomass positively correlated with the root biomass at both the community and functional group levels (Figure 3D), while the relationship between the R/S ratio and the shoot biomass or root biomass was non-significant (Figures 3E,F).

### Effects of Land Degradation on Plant Aboveground Traits and Root Competition

**FIGURE 4:**
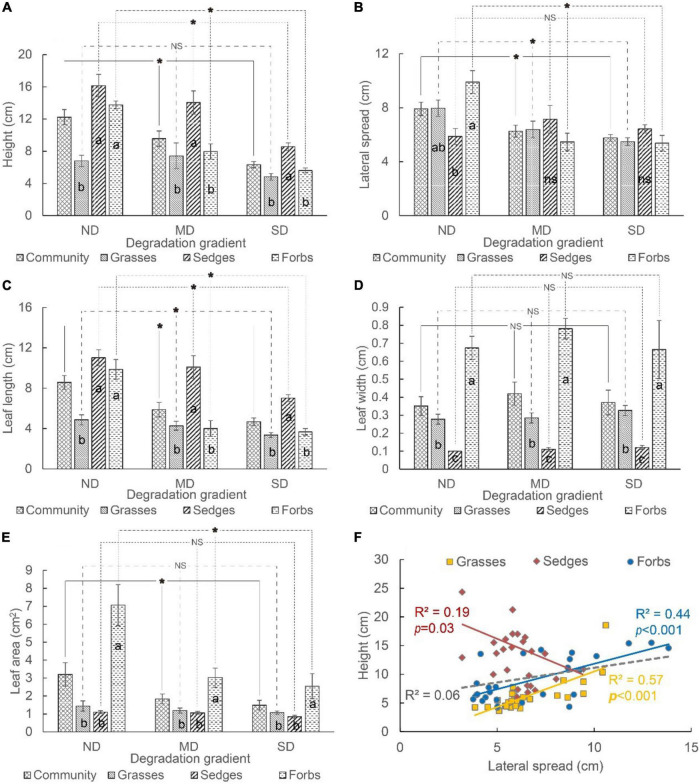
The **(A)** height, **(B)** lateral spread, **(C)** leaf length, **(D)** leaf width, and **(E)** leaf area of different functional groups (i.e., grasses, sedges, and forbs) in different degradation gradients. The relationship between plant height and lateral spread varied among different functional groups **(F)**. The “*” means a significant difference between functional groups among degradation gradients. The different lowercase letters mean significant differences between functional groups in each degradation gradient. “NS” and “ns” mean no differences.

For the whole community, the average height, lateral spread, leaf length, and leaf area decreased, but the leaf width did not change along the degradation gradient (Figures 4A–E). For grasses, the lateral spread and leaf length decreased, but the height, leaf width, and leaf area did not change along the degradation gradient. For sedges, the height and leaf length decreased, while the lateral spread, leaf width, and leaf area did not change along the degradation gradient. For forbs, all traits except leaf width decreased along the degradation gradient (Figures 4A–E).

Most aboveground traits of grasses, sedges, and forbs were different at each degradation level. The height and leaf length of these three functional groups showed the same pattern, i.e., height and leaf length of sedges and forbs were higher than that of grasses in ND; the height and leaf length of sedges were higher (or larger) than that of grasses and forbs in MD and SD (Figures 4A,C). The lateral spread of forbs was higher than that of sedges but did not vary with that of grasses in ND. The lateral spread did not differ among grasses, sedges, and forbs in MD and SD (Figure 4B). The order of leaf width was forbs > grasses > sedges in ND, MD, and SD (Figure 4D). The leaf area of forbs was higher than that of grasses and sedges. The leaf area of grasses and sedges did not vary significantly in each degradation level (Figure 4E).

A non-significant correlation between the lateral spread and height was tested at the community level. It was a positive correlation between these two indicators for grasses and forbs, while it was a negative correlation for sedges (Figure 4F).

**FIGURE 5:**
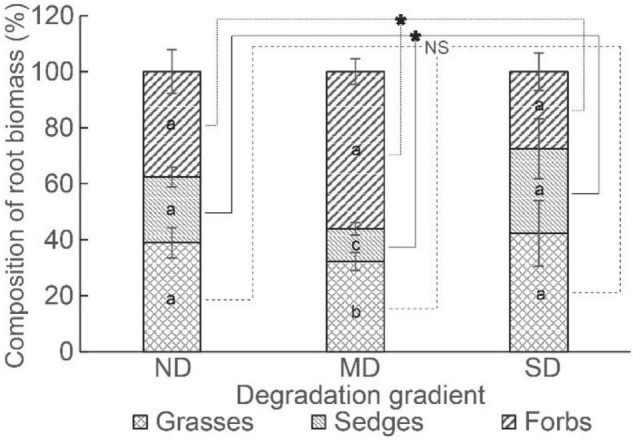
The root competition in different degradation gradients. The “*” means a significant difference among degradation gradients. Different lowercase letters mean significant differences between functional groups in each degradation gradient.

The root competition (i.e., the percentage of root biomass in the gross root biomass of a functional group) of grasses, sedges, and forbs was similar in ND and SD. In MD, the root competition was ordered as forbs > grasses > sedges (Figure 5). The root competition of grasses did not vary among degradation levels, while the root competition of sedges was the lowest and the root competition of forbs was the highest in MD (Figure 5).

### Linkages Among Aboveground Traits, Root Competition, and Biomass Allocation

**FIGURE 6:**
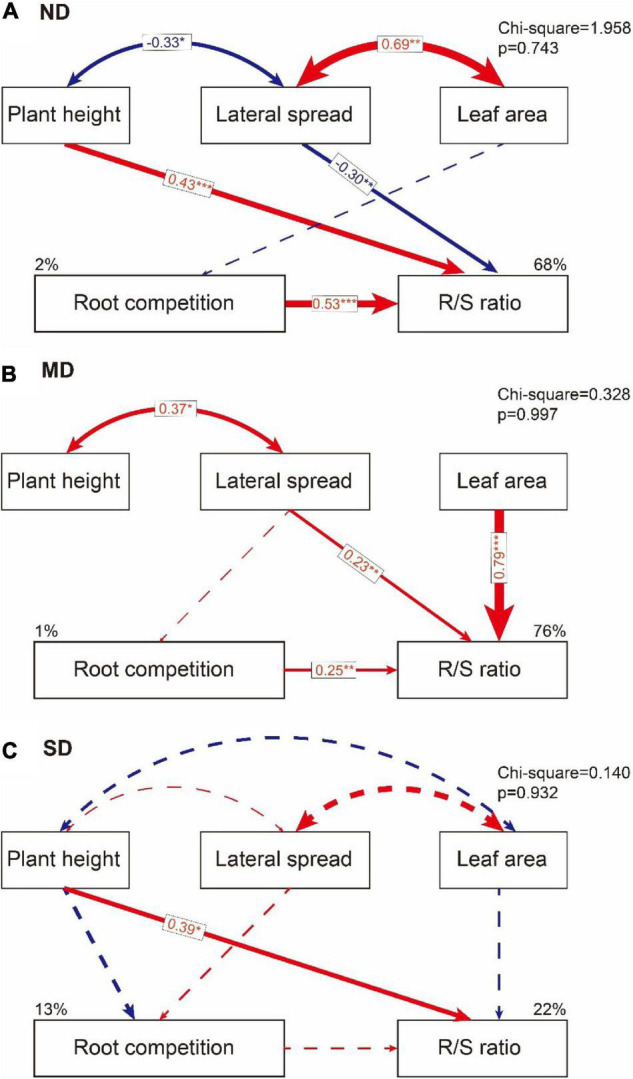
The direct and indirect effects of plant height, lateral spread, and leaf area on root competition and R/S ratio in **(A)** no obvious degradation (ND) plots, **(B)** moderate degradation (MD) plots, and **(C)** severe degradation (SD) plots. The blue line means negative effects and the red line means positive effects. The solid line means significant path (*p < 0.1; **p < 0.05; ***p < 0.01). The dashed line means a non-significant path that helped to improve model quality.

The effects of aboveground traits and root competition on the R/S ratio varied among different degradation levels. In ND plots, plant height and root competition positively affected the R/S ratio, while the lateral spread negatively affected the R/S ratio in a direct path. The leaf area weakly affected the R/S ratio through the lateral spread and root competition (Figure 6A and Table 3). In MD plots, the R/S ratio was positively affected by the lateral spread, leaf area, and root completion in a direct path. The plant height affected the R/S ratio through the lateral spread (Figure 6B and Table 3). In SD plots, the R/S ratio was positively affected by plant height in a direct path. The direct effects of lateral spread, leaf area, and root competition on the R/S ratio were weak (Figure 6C and Table 3).

**TABLE 3:** Standardized total effects of plant height, lateral spread, leaf area, and root competition on R/S ratio in different degradation gradients in an alpine meadow in the eastern Qinghai-Tibetan Plateau.

Treatment		Height	Lateral spread	Leaf area	Root competition
ND	Root competition	/	0.44	−0.14	/
	R/S ratio	0.43	−0.30	−0.08	0.53
MD	Root competition	/	0.11	/	/
	R/S ratio	/	0.26	0.79	0.25
SD	Root competition	−0.33	0.18	/	/
	R/S ratio	0.33	0.03	−0.21	0.19

## Discussion

Generally, a considerable fraction of plant biomass distributes in belowground in grassland ecosystems (Jackson et al., 1997; Peng et al., 2020). It is always a positive relationship between the root and shoot biomass (Mokany et al., 2006; Chen et al., 2019). We got the same result at both the community and functional group levels in this study.

The R/S ratio is strongly affected by the root and shoot biomass. The plant root is the fundamental structure for plants to uptake water and nutrients and plays an important role in carbon sink in grassland ecosystems (Jackson et al., 1997; Pausch and Kuzyakov, 2018). However, the root biomass is hard to be evaluated accurately (Ma et al., 2021). The quadrat-scale sampling (usually 1 m × 1 m) for shoot biomass and the soil core-scale sampling (usually with a diameter of 5–10 cm) for root biomass were always used in field surveys to investigate the shoot and root biomass in grassland ecosystems (Yang et al., 2010). This asynchronous sampling, i.e., the different locations of the shoot biomass and root biomass, might cause significant effects on the R/S ratio calculation. Meanwhile, it is inaccurate to select the live root from soil cores according to its properties (such as color and attached fine roots), which might misestimate the R/S ratio as well. In this study, the integrated shoot and root sampling method was used, which could improve the accuracy of the estimation of the R/S ratio. Based on these matched biomass data, we found that it was hard to directly predict the R/S ratio according to the shoot biomass or root biomass.

Plants should invest more energy to uptake nutrients and water from the soil to adapt to harsh habitats (Dai et al., 2020). Consequently, the R/S ratio tends to be high (always >1) in some biomes (e.g., tundra and grassland) distributed in cold and dry habitats (Jackson et al., 1996; Poorter et al., 2012). In this study, we found that the R/S ratio of all meadow plants is >2 at both community level and functional group level under the land degradation conditions. Furthermore, the R/S ratio of plant community did not vary among degradation gradients because the shoot biomass decreased but the root biomass did not change along the degradation gradient. This phenomenon was consistent with findings obtained by other studies (Zhang et al., 2019). Root competition would decrease if soil nutrient levels increased (Casper and Jackson, 1997). Contrarily, grassland degradation, which always decreases soil nutrients (Liu et al., 2020), would improve soil nutrient limitation and might increase root competition. Therefore, the little change of root biomass in the present study might be obligated to the intensification of root competition.

At the functional group level, we found that the root biomass of grasses, sedges, and forbs did not vary in ND and SD, but it was ordered as forbs > grasses > sedges in MD. This phenomenon could be explained by the moderate interference theory. The R/S ratio of grasses and forbs did not change; however, the R/S ratio of sedges increased along the degradation gradients. This result revealed that the change in the R/S ratio caused by land degradation might be considered more at the functional group level rather than the community level in alpine meadow ecosystems.

The factors that affect the R/S ratio are widely discussed (Yang et al., 2018). Some evidence suggests that root interactions could affect the allocation pattern of plant biomass (Poorter et al., 2012). Especially, from the competition view, it suggests that root competition is always more important than shoot competition in affecting the performance of plant survival (Donald, 1958; Wilson, 1988). In this study, we found that the effects of root competition on the R/S ratio were always positive and linearly decreased along the degradation gradients. The total effects of shoot competition (including vertical and horizontal competition) on the R/S ratio were positive but showed a non-linear decrease along the degradation gradients because the vertical competition (presented by the plant height) and horizontal competition (presented by lateral spread and leaf area) played different roles in regulating the R/S ratio at different degradation conditions. When the meadow was healthy (e.g., ND in this study) or severely degraded (e.g., SD in this study), the vertical and horizontal competition played opposite roles, i.e., the vertical competition promoted the R/S ratio, while the horizontal competition reduced it. However, when it was a moderate degradation (e.g., MD in this study), the horizontal competition promoted the R/S ratio, but the vertical competition did not affect it. These results suggested that the first hypothesis of this study was denied, while the second hypothesis was confirmed.

In addition to biotic factors, climatic conditions could strongly affect the R/S ratio of plants, e.g., the cold and dry conditions tended to a high R/S ratio (Reich et al., 2014; Ma et al., 2021). But the R/S ratio is hard to be estimated by climatic indicators (e.g., temperature and precipitation) (Yang et al., 2010). Therefore, the indirect-observed R/S ratio inferred according to climatic indicators, which may occur in large-scale studies, should be cautiously used.

## Conclusion

The root competition promoted the R/S ratio, and this process linearly decreased with land degradation gradients. The shoot competition promoted the R/S ratio as well, but the effects of shoot competition on the R/S ratio showed a non-linear decrease along the degradation gradients because the vertical and horizontal competition always played opposite roles in affecting the R/S ratio. These results indicate that plant competition might be a critical factor to explain why degraded alpine ecosystem has a high R/S ratio.

## Data Availability Statement

The original contributions presented in the study are included in the article/Supplementary Material, further inquiries can be directed to the corresponding author.

## Author Contributions

YZ designed the experiment. QZ, YM, KL, HY, KW, and XW conducted the field survey and laboratorial works. YZ, XG, and XH wrote the manuscript, the other co-authors discussed and improved the manuscript. All authors contributed to the article and approved the submitted version.

## Conflict of Interest

The authors declare that the research was conducted in the absence of any commercial or financial relationships that could be construed as a potential conflict of interest.

## Publisher’s Note

All claims expressed in this article are solely those of the authors and do not necessarily represent those of their affiliated organizations, or those of the publisher, the editors and the reviewers. Any product that may be evaluated in this article, or claim that may be made by its manufacturer, is not guaranteed or endorsed by the publisher.
