# Supplementary material for: Land Degradation Changes the Role of Above- and Belowground Competition in Regulating Plant Biomass Allocation in an Alpine Meadow
Source: Front Plant Sci. 2022 Feb 3;13:822594. doi: 10.3389/fpls.2022.822594 (PMC8850915; doi:10.3389/fpls.2022.822594)
Supplement: Supplementary file 1 [file Data_Sheet_1.pdf]

Land degradation changes the role of above- and below-ground competition in regulating plant biomass allocation in an alpine meadow

Yong Zhang[1, 2, *], Qiuzhu Zheng[1, 2], Xiaoxia Gao[3], Yandan Ma[1, 2], Kemin Liang[1, 2], Haitao Yue[1, 2], Xiaoxia Huang[4], Kaiting Wu[1, 2], Xiaorong Wang[1, 2]

1Yunnan Key Laboratory of Plateau Wetland Conservation, Restoration and Ecological Services, College of Wetlands, Southwest Forestry University, Kunming 650224, P. R. China

2 National Plateau Wetlands Research Center, Southwest Forestry University, Kunming 650224, P. R. China

3 State Key Laboratory of Vegetation and Environmental Change, Institute of Botany, Chinese Academy of Sciences, Beijing 100093, P. R. China

4 School of Earth Sciences, Yunnan University, Kunming 650091, Yunnan, P. R. China

[*] *Corresponding author. E-mail: zhy1902@126.com*

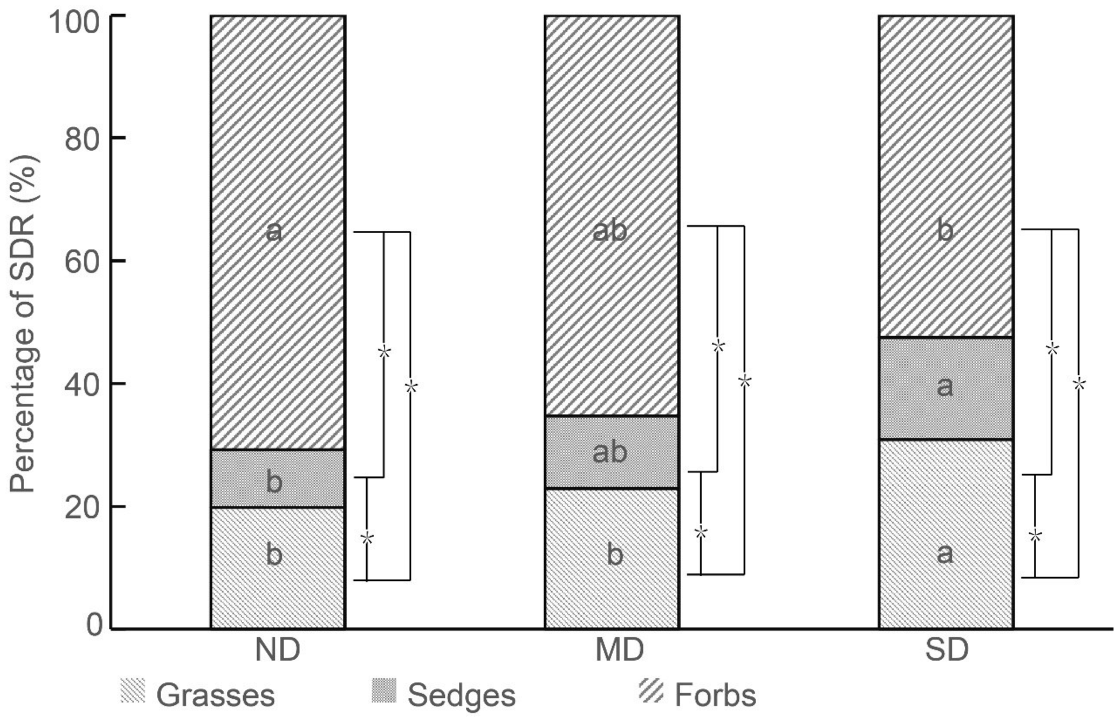

Figure S1 The composition of functional groups (i.e., grasses, sedges and forbs) in different degradation gradients. The “*” means significant difference between functional groups in each degradation gradient. The different lowercase letters mean significant differences between each functional group among degradation gradients.

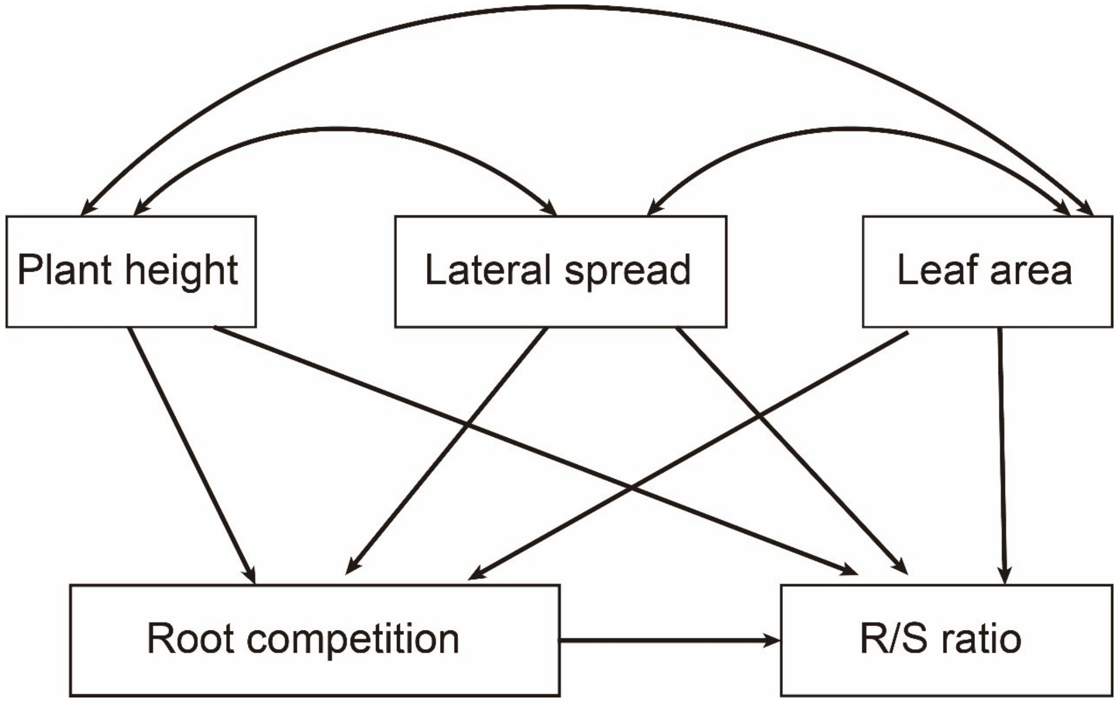

Figure S2 A *priori* model for the effects of plant aboveground morphological traits on root competition and R/S ratio.

Table S1 Model selection procedure and statistics for structure equation model explaining R/S ratio of no obvious degradation (ND) in Fig. 4a. The best model was marked in bold.

	Stepwise removal of non-significant paths	*Df*	*AIC*	χ^2	*p*	*CFI*	*TLI*	*RMSEA*	*Squared Multiple Correlations of endogenous variable*	
									Root competition	R/S ratio
A *priori* model		0	40.000	0.000	—	1.000	—	0.365	0.126	0.607
Model 1	Plant height ↔ Leaf area	1	38.017	0.017	0.895	1.000	1.433	<0.001	0.124	0.611
Model 2	Leaf area→R/S ratio	2	36.019	0.019	0.990	1.000	1.437	<0.001	0.124	0.611
Model 3	Plant height→Root competition	3	34.075	0.075	0.995	1.000	1.430	<0.001	0.122	0.621
**Model 4**	**Lateral spread→Root competition**	**4**	**33.958**	**1.958**	**0.743**	**1.000**	**1.225**	**<0.001**	**0.020**	**0.675**
Model 5	Leaf area→Root competition	5	32.303	2.303	0.806	1.000	1.238	<0.001	0.000	0.665

Table S2 Model selection procedure and statistics for structure equation model explaining R/S ratio of moderate degradation (MD) in Fig. 4b. The best model was marked in bold.

	Stepwise removal of non-significant paths	*Df*	*AIC*	χ^2	*p*	*CFI*	*TLI*	*RMSEA*	*Squared Multiple Correlations of endogenous variable*	
									Root competition	R/S ratio
A *priori* model		0	40.000	0.000	—	1.000	—	0.334	0.016	0.750
Model 1	Plant height ↔ Leaf area	1	38.027	0.027	0.869	1.000	1.379	<0.001	0.015	0.752
Model 2	Leaf area↔ Lateral spread	2	36.044	0.044	0.978	1.000	1.381	<0.001	0.015	0.754
Model 3	Plant height→Root competition	3	34.047	0.047	0.997	1.000	1.383	<0.001	0.015	0.754
Model 4	Plant height→R/S ratio	4	32.221	0.221	0.994	1.000	1.368	<0.001	0.015	0.752
**Model 5**	**Leaf area→Root competition**	**5**	**30.328**	**0.328**	**0.997**	**1.000**	**1.364**	**<0.001**	**0.011**	**0.759**
Model 6	Lateral spread→Root competition	6	28.581	0.581	0.997	1.000	1.352	<0.001	0.000	0.756

Table S3 Model selection procedure and statistics for structure equation model explaining R/S ratio of severe degradation (SD) in Fig. 4c. The best model was marked in bold.

	Stepwise removal of non-significant paths	*Df*	*AIC*	χ^2	*p*	*CFI*	*TLI*	*RMSEA*	*Squared Multiple Correlations of endogenous variable*	
									Root competition	R/S ratio
A *priori* model		0	40.000	0.000	—	1.000	—	0.133	0.127	0.224
Model 1	Plant height ↔ Lateral spread	1	38.192	0.192	0.662	1.000	3.875	<0.001	0.138	0.224
Model 2	Plant height ↔ Leaf area	2	38.358	2.358	0.308	0.872	0.362	0.106	0.141	0.183
Model 3	Plant height ↔ Lateral spread	3	40.586	6.586	0.086	0.000	-3.252	0.273	0.144	0.210
Model 4	Leaf area→Root competition	4	38.590	6.590	0.159	0.079	-1.303	0.201	0.138	0.209
Model 5	Lateral spread→R/S ratio	5	36.726	6.726	0.242	0.386	-0.228	0.147	0.138	0.191
Model 6	Lateral spread→Root competition	6	35.275	7.275	0.296	0.546	0.244	0.115	0.096	0.193
Model 7	Root competition→R/S ratio	7	33.924	7.924	0.339	0.671	0.530	0.091	0.096	0.156
Model 8	Leaf area→R/S ratio	8	32.596	8.596	0.378	0.788	0.735	0.068	0.096	0.150
Model 9	Plant height→Root competition	9	32.214	10.214	0.333	0.568	0.52	0.092	0.000	0.150
Model 10*	Leaf area→Root competition	1	38.004	0.004	0.947	1.000	4.541	<0.001	0.127	0.223
**Model 11***	**Lateral spread→R/S ratio**	**2**	**36.140**	**0.140**	**0.932**	**1.000**	**4.308**	**<0.001**	**0.127**	**0.216**
Model 12*	Lateral spread→Root competition	3	34.690	0.690	0.876	1.000	3.739	<0.001	0.096	0.222
Model 13*	Root competition→R/S ratio	4	33.339	1.339	0.855	1.000	3.367	<0.001	0.096	0.185

*: Model was re-optimized from a *priori* model.
